# Wall stretch sign to prevent trans-gastric lumen apposing metal stent deployment during endoscopic ultrasound-guided gallbladder drainage

**DOI:** 10.1055/a-2873-7567

**Published:** 2026-06-01

**Authors:** Haruo Miwa, Kazuki Endo, Ryo Soma, Ritsuko Oishi, Yuichi Suzuki, Hiromi Tsuchiya, Shin Maeda

**Affiliations:** 1 Gastroenterological Center 26437 Yokohama City University Medical CenterYokohamaJapan; 2 Department of GastroenterologyYokohama City University Graduate School of MedicineYokohamaJapan

**Fig. 1:**
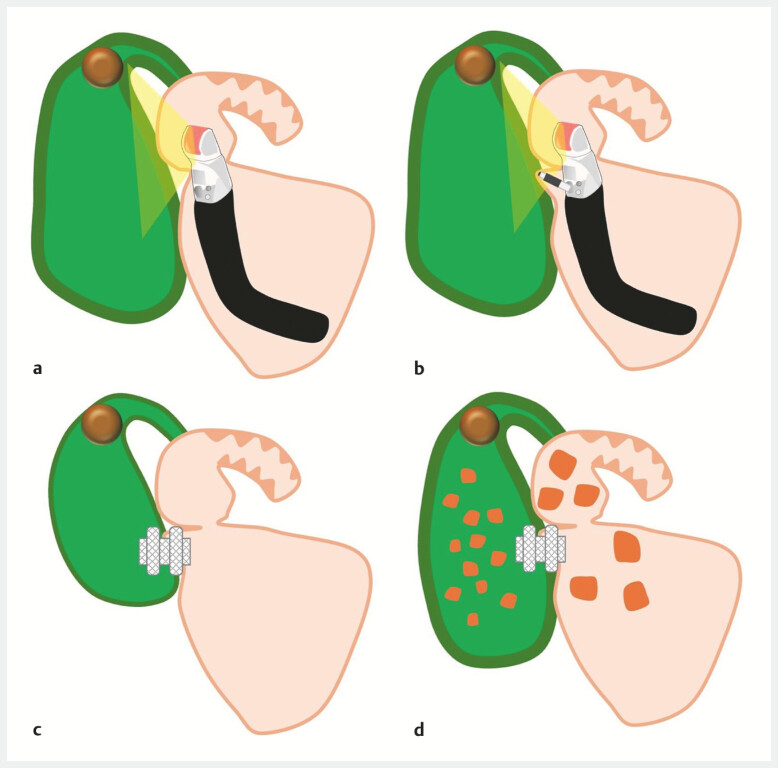
Schematic illustration of the transgastric placement of a lumen-apposing metal stent (LAMS) during endoscopic ultrasound-guided gallbladder drainage. **a** The echoendoscope transducer is advanced into the duodenum. **b** The working channel outlet remains within the stomach. **c** The LAMS is placed in the gastric antrum. **d** Recurrent cholecystitis occurs due to food residues entering the gallbladder.

Endoscopic ultrasonography-guided gallbladder drainage (EUS-GBD) using a lumen-apposing metal stent (LAMS) is an established alternative for acute cholecystitis in patients unfit for surgery 1 2 . Although a transduodenal approach is generally preferred to reduce recurrent cholecystitis caused by food residue entering the gallbladder ( Fig. 1 , 3 ), confirming true duodenal positioning can be difficult when the EUS transducer is advanced into the duodenum while the working channel outlet remains within the stomach. In such situations, advancement of the LAMS delivery system causes the visible stretching of the gastric wall on EUS. We defined this finding as the “wall stretch sign.” Here, we describe a simple technique using the wall stretch sign to confirm whether the working channel outlet is located in the stomach or duodenum during EUS-GBD ( Video 1 ).

The wall stretch sign is useful to confirm whether the working channel outlet is located in the stomach or duodenum during EUS-GBD. EUS-GBD, Endoscopic ultrasonography-guided gallbladder drainage.Video 1

**Fig. 2:**
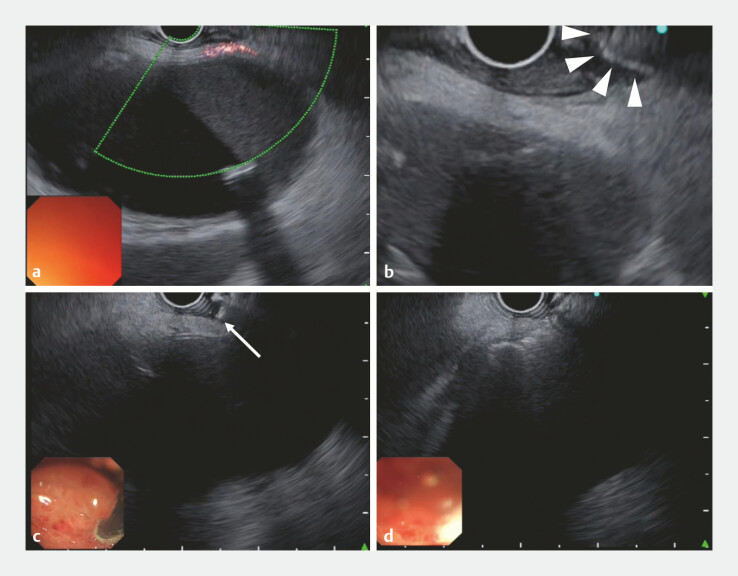
Case 1. **a** An ultrasound image shows a swollen gallbladder. **b** The wall stretch sign is visible when the LAMS delivery system is exposed from the stomach (arrowheads). **c** The echoendoscope is advanced further into the duodenum bulb. The LAMS delivery system is re-exposed (arrow). **d** The LAMS is safely deployed. LAMS, lumen-apposing metal stent.

In Case 1, although the echoendoscope (GF-UCT260, Olympus, Tokyo, Japan) was advanced into the duodenal bulb and the gallbladder was visualized, the working channel outlet remained in the stomach. Advancement of the LAMS delivery system (Hot AXIOS system 10mm, Boston Scientific, Marlborough, MA, US) demonstrated the wall stretch sign, indicating gastric positioning. After further scope advancement into the duodenal bulb, the LAMS was safely deployed ( Fig. 2 ).

**Fig. 3:**
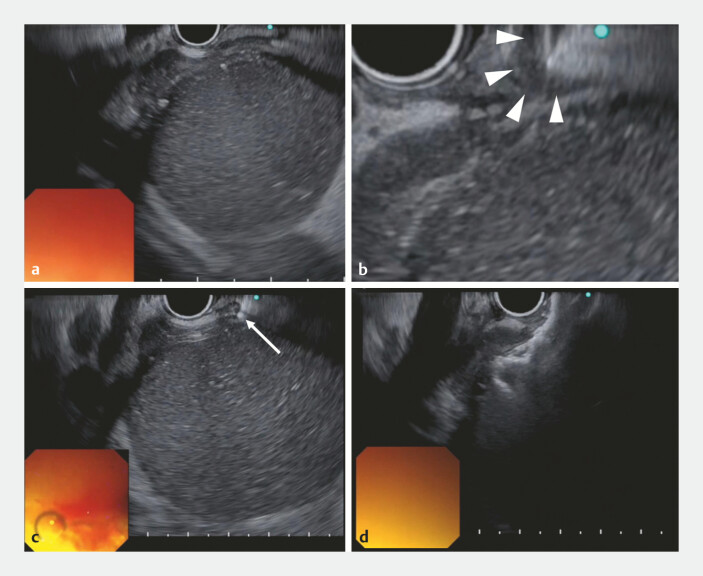
Case 2. **a** An enlarged gallbladder filled with debris is visualized on EUS. **b** Advancement of the LAMS delivery system demonstrates the wall stretch sign (arrowheads). **c** After advancing the echoendoscope into the duodenum, the wall stretch sign is no longer observed (arrow). **d** The LAMS is deployed in the standard manner. EUS, endoscopic ultrasound; LAMS, lumen-apposing metal stent.

In Case 2, an enlarged gallbladder filled with debris was visualized on EUS from the duodenal bulb. When the LAMS delivery system was pushed against the enteral wall, the wall stretch sign was observed. The echoendoscope was then advanced into the second portion of the duodenum. The LAMS delivery system was re-exposed. As the wall stretch sign was no longer observed, the delivery system was advanced electrocautery-assisted into the gallbladder. The LAMS was deployed in the standard manner from the duodenal bulb ( Fig. 3 ).

To the best of our knowledge, this is the first report describing the wall stretch sign as an indicator that the working channel outlet of the echoendoscope remains within the stomach during EUS-GBD.

Endoscopy_UCTN_Code_TTT_1AS_2AK
